# Effect of individualized PEEP titrated by EIT on postoperative atelectasis in children undergoing laparoscopy: A randomized controlled trial

**DOI:** 10.7150/ijms.112280

**Published:** 2025-06-12

**Authors:** Sheng-Hua Wang, Ye Wang, Si-Yuan Li, Lai Jiang, Yan-Fei Mao, Qin Xia, Han Gan

**Affiliations:** 1 Department of Anesthesiology and Surgical Intensive Care Unit, Xinhua Hospital Affiliated to Shanghai Jiao Tong University School of Medicine, 200092, Shanghai, China.; 2 Clinical Medical School, Xinhua Hospital Affiliated to Shanghai Jiao Tong University School of Medicine, 200092, Shanghai, China.

**Keywords:** pulmonary atelectasis, electrical impedance tomography, PEEP titration, children

## Abstract

**Background:** Atelectasis is common during perioperative period in children. Although positive end positive end-expiratory pressure (PEEP) has been identified as a lung protective ventilation strategy to alleviate atelectasis, there's still no consensus on the optimal value of PEEP. We hypothesized that individualized PEEP titrated by electrical impedance tomography (EIT) may reduce the incidence of postoperative atelectasis.

**Methods:** A total of 50 children aged between 2 to 7, undergoing laparoscopic hernia repair, were randomly divided into two groups according to the principle of randomization: a control group (PEEP5) and an experimental group (EIT). In the control group, PEEP was set to a fixed value of 5mmHg during pneumoperitoneum mechanical ventilation. The EIT group received an individualized PEEP determined by a decremental PEEP titration using EIT. Ultrasonic assessment and score of atelectasis were carried out post-intubation, post-surgery, and one hour post-extubation. For this study, a total of 12 lung regions were evaluated by Lung ultrasonography, and significant atelectasis was defined by a consolidation score of at least 2 in any region. The primary outcome was the incidence of atelectasis at post-surgery.

**Results:** The incidence of atelectasis after surgery was 92% in the control groups (n=25) and 64% in the EIT groups (n=25), respectively (odds ratio [OR], 0.72; 95% confidence interval [CI], 0.094 to 4.827; P = 0.037). The incidence of atelectasis after 1h post-extubation was 80% in the control groups (n=20) and 48% in the EIT groups (n=12), respectively (odds ratio [OR], 0.59; 95% confidence interval [CI], 0.149 to 2.215; P = 0.038). Both lung consolidations and B-lines score were significantly higher in control group than in the EIT group after surgery (consolidations: 9 vs. 7, P = 0.027; B-lines: 11 vs. 8, P = 0.002) and 1h post-extubation (consolidations: 7 vs. 4, P = 0.018; B-lines: 7 vs. 5, P = 0.037). Lung compliance using optimal PEEP during mechanical ventilation was 20.0±3.3 ml/cm H_2_O. The desaturation (pulse oximeter value is below 95%) after extubation was observed in 7 in the control group and 1 in the EIT group (P = 0.048). Hemodynamics were stable during titration.

**Conclusion:** EIT-directed individualized PEEP titration can reduce the incidence and severity of postoperative atelectasis in children undergoing laparoscopic surgery.

## Introduction

Atelectasis frequently occurs as a complication during the perioperative period due to general anesthesia.^1^ During laparoscopic surgery, pneumoperitoneum leads to high intra-abdominal pressures which can result in cephalad displacement of the diaphragm and reduced lung compliance, potentially exacerbating the atelectasis.^2^-^4^ This issue can get worse in children because of their unique physiology , such as high pulmonary closing capacity, hypoxaemia, immature lung parenchyma.^5^,^6^ Pulmonary atelectasis can adversely affect gas exchange and respiratory mechanics, potentially leading to postoperative postoperative respiratory insufficiency and worse clinical outcomes.^4^ PEEP has been shown to improve gas exchange and reduce the area of atelectasis in children.^7^ It has been proposed to be one of the lung protective ventilation strategies, usually not exceeding 5 cm H_2_O.^8^ However, setting a fixed PEEP may not be efficient due to individual physiological differences among children during general anesthesia.^9^-^11^

Electrical Impedance Tomography (EIT), a non-invasive and bedside technique, is now utilized to detect the dynamic regional distribution of lung aeration. Of note, recent studies have highlighted its potential as a means to determine the individual PEEP value during intraoperative lung ventilation.^12^-^14^ Lung ultrasound (LUS), another convenient and radiation-free tool, has shown the accuracy in monitoring and diagnosing atelectasis using lung ultrasound score in children through a growing body of researches these years,^15^,^16^ making it especially suitable for those undergoing general anesthesia with lung ventilation.

The aim of our study was to assess the influence on atelectasis of optimal PEEP titrated by EIT in children undergoing laparoscopic hernia repair. We mainly hypothesized that the incidence of atelectasis after the surgery in children using personalizing PEEP would be lower compared to those with a fixed PEEP in control group.

## Material and Methods

### Ethics approval and registration

This study was approved by the ethics committee of Xinhua Hospital Affiliated to Shanghai Jiao Tong University School of Medicine, Shanghai, China on 3 February 2023 (approval number: XHEC-C-2023-008-1). The trial was registered on https://www.chictr.org.cn/showproj.html?proj=178992 (trial number: ChiCTR 2300068208). The study was conducted following the Consolidated Standards of Reporting Trials (CONSORT) guidelines.^17^

### Participants

The study was performed between 12 February 2023 and 30 April at Xinhua Hospital affiliated to Shanghai Jiao Tong University School of Medicine. Participants included children aged 2 to 7 years who were undergoing laparoscopic surgery and had an American Society of Anesthesiologists (ASA) physical status of I or II. Parental consent was obtained prior to their inclusion in the study. The study excluded children with hemodynamic instability; those with abdominal distension or apparent lung, heart, or chest wall diseases; obese or overweight children; and children whose operation time exceeded 2 hours. Additionally, patients who required an intraoperative change in the mode were also excluded.

### Randomization and blinding

Following a simple randomisation procedure (computerised random number; https://www.randomizer.org), an anaesthetic assistant who was not involved in the study prepared sealed opaque envelopes and randomly allocated the enrolled patients to the control group or the EIT group in an allocation ratio of 1:1. An experienced anesthesiologist unsealed opaque envelopes, and was responsible for EIT titration and anaesthesia maintenance. Additionally, the children, guardians and the outcome assessor who performed lung ultrasound were blinded to the group allocation.

### Study design and anesthesia protocol

Prior to the induction of anesthesia, all children were connected with monitoring devices, including ECG, noninvasive arterial pressure, and pulse oximetry. Following pre-oxygenation with 100% oxygen, anesthesia was induced with propofol 3 mg•kg^-1^, rocuronium bromide 0.6 mg•kg^-1^, fentanyl 1µg•kg^-1^. Anesthesia was maintained with sevoflurane 0.8-1.0 MAC, propofol 4 mgkg^-1^h^-1^and remifentanil 0.01 µg•kg^-1^•min^-1^ infusion. Mechanical ventilation settings were specified as follows: volume-controlled mode with a tidal volume of 8 ml•kg^-1^; a fraction of inspired oxygen of 0.5; an inspiration-to-expiration ratio of 1:2. The respiratory rate was set from 18 to 22 breaths min^-1^ in order to keep end-tidal carbon dioxide concentration at 35-45 mmHg. Pneumoperitoneum pressure was set at 8 mmHg.

Connection with monitoring devices occurred when they entered the operation room. In control group, recruitment manoeuvre was performed two minutes after pneumoperitoneum and a PEEP of 5 cm H_2_O was applied with the same ventilation settings as we mentioned before during the ventilation. While the EIT group maintained a PEEP of 5 cm H_2_O after intubation until decremental PEEP titration was implemented 2 minutes after the start of pneumoperitoneum. During our entire process, our primary outcome target was the incidence of atelectasis after surgery. The hyperventilation area included the incidence of atelectasis after 1h post-extubation, the lung ultrasound score after surgery and after 1h post-extubation, the lung compliance, the hemodynamic parameters (mean arterial pressure, heart rate); SpO2 reduction in PACU (<95%); incidence of hemodynamic impairment.

### PEEP titration

**Figure 1:**
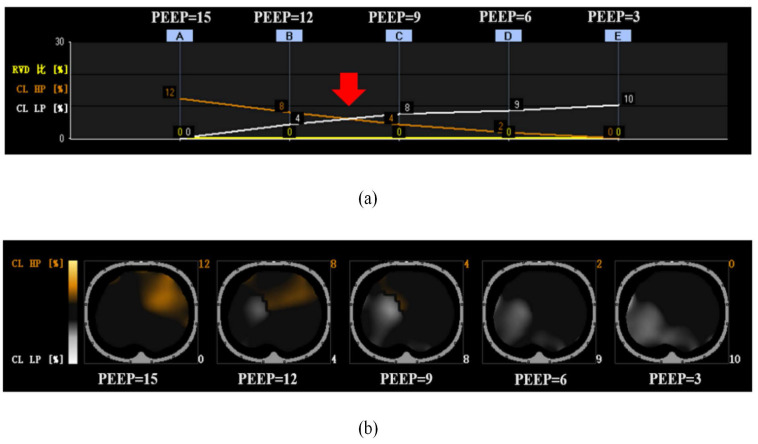
One case in EIT group of decremental PEEP titration, according to the method originally described by Costa *et al.* (a) The individualized PEEP for this case: Red arrow point. (b) The orange line is the estimate of overdistended lung, was gradually decreased with the decrease of PEEP titration. The white line is the estimate of total collapsed lung, mainly in the dorsal. EIT, Electrical impedance tomography; PEEP, positive end-expiratory pressure.

We attached the EIT electrode around the skin of the children in the EIT group at the level of fourth rib and connected to an EIT monitor (PulmoVista® 500, Dräger, Lübeck, Germany) before anesthesia induction. The process was initiated by performing a lung recruitment manoeuvre in pressure-controlled mode, as outlined by Costa *et al.*^18^ This involved maintaining a steady airway pressure of 15 cm H_2_O, with 5 cm H_2_O increments in PEEP until a peak inspiratory pressure of 30 cm H_2_O which was maintained for 10 seconds. Each PEEP level was maintained for 5 seconds. 2 minutes after the onset of pneumoperitoneum, following this, we implemented a decremental PEEP titration plan, starting from a level of 15 cm H_2_O and decreasing in steps of 3 cm H_2_O every 90 seconds until we reached 3 cm H_2_O. The EIT monitor could create a graph of the percentage of collapse and overdistension at each PEEP when titration was finished. We identified the optimal PEEP as the intersection on the EIT curves, representing the most balanced compromise between lung collapse and overdistension (Fig. 1,a).^18^ Children in the EIT group respectively applied the optimal PEEP when it had been determined until the end of pneumoperitoneum. Following this, we reapplied a PEEP of 5 cm H_2_O to the EIT group, maintaining this until the end of mechanical ventilation. Vital signs and pulmonary compliance were closely monitored and recorded during titration.

### Lung ultrasound

According to Acosta *et al.*, each hemithorax was divided into 6 regions using three longitudinal lines (parasternal, anterior and posterior axillary) and two axial lines (above the diaphragm and 1 cm above the nipples).^15^ The degree of consolidation and B-lines were evaluated and scored for each region. Each sign was scored from 0 to 3. Lung consolidations: 0, no consolidation; 1, minimal juxtapleural consolidation; 2, small-sized consolidation; and 3, large-sized consolidation. B-lines: 0, fewer than three isolated B-lines; 1, multiple and well-defined B-lines; 2, multiple coalescent B-lines; and 3, white lung. The lung ultrasound score was defined by the sum of the B-line score and the juxtapleural consolidation score in 12 regions and ranged from 0 to 36. We defined significant atelectasis as a consolidation score of at least 2 in any region. Lung ultrasound assessments were conducted three times: after intubation (T1), after surgery (T2), and after 1h post-extubation (T3).

### Statistical analysis

A previous study^19^ showed that the incidence of atelectasis was 94% in children undergoing general anesthesia using magnetic resonance examination. Compared to magnetic resonance examination, LUS demonstrated an 88% sensitivity in diagnosing atelectasis.^15^ In this study, we recruited children undergoing laparoscopic surgery using pneumoperitoneum which could aggravate alveolar collapse through increased abdominal pressure, thus increasing the incidence of atelectasis. Therefore, we assumed that the incidence of atelectasis in control group at T2 was 90%, while that in EIT group was 50%, assuming a beta-power of 80% and an alpha-error of 5%. According to the sample loss of 20%, the required sample size was eventually set at 50, 25 per group.

Statistical analysis was performed using IBM SPSS Statistics version 20. The Kolmogorov-Smirnov test was used to evaluate the normality of the measurement data. Measurement data conforming to normal distribution were expressed as mean ± standard deviation (*x*±*s*), and two independent samples T-test was used for comparison between groups. Measurement data with non-normal distribution were expressed as median (M) and interquartile distance (IQR), and Mann-Whitney U test was used for comparison between groups. Count data were expressed as n (%), and comparisons between groups were made using Chi-square test or Fisher exact probability method. Repeated measures data were analyzed using repeated measures analysis of variance. A P value less than 0.05 was considered statistically significant.

## Results

**Figure 2:**
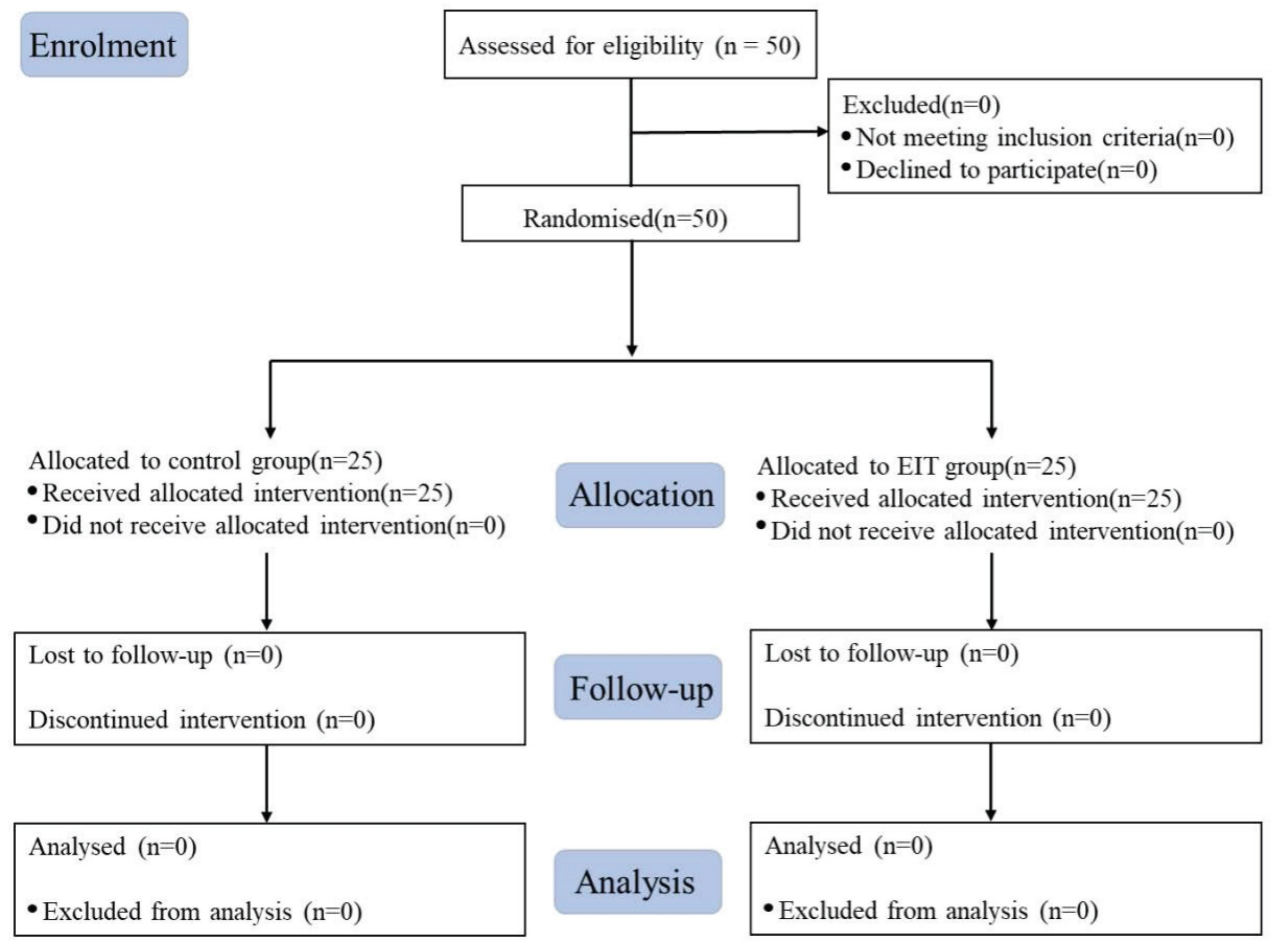
Study flow chart.

A total of 50 children were randomised into the control (n =25) and EIT (n =25) groups between 12 February 2023 and 30 April at Xinhua Hospital affiliated to Shanghai Jiao Tong University School of Medicine. No one was excluded from each group. Eventually, 50 children were analyzed (Fig. 2). Table 1 demonstrates the similar baseline characteristics of the study cohort.

**Figure 3:**
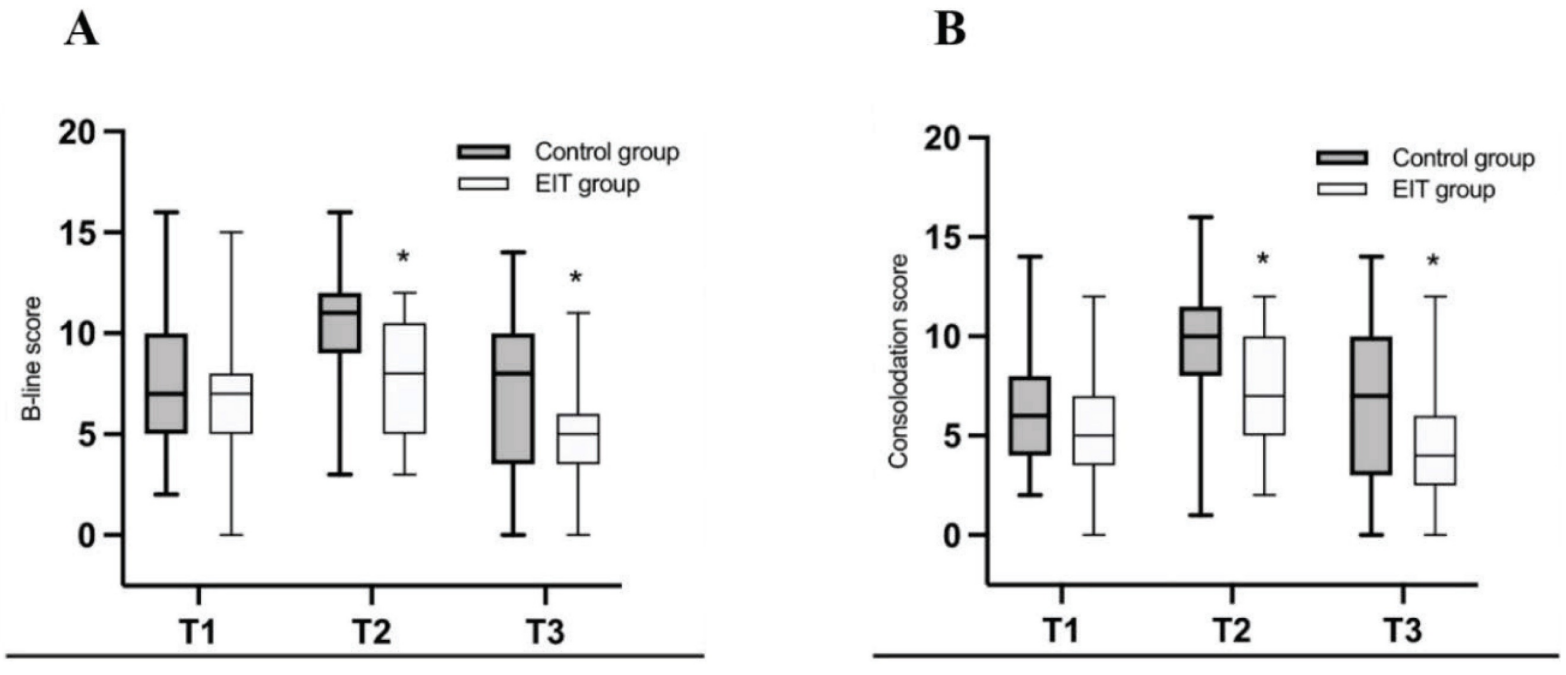
B-line scores (a) and Consolidation scores (b) from T1 to T3 control group, grey box; EIT group, white box. *P<0.05.

With regard to the primary outcome, the incidence of atelectasis after surgery was significantly higher in the control groups (92%) than EIT (64%, OR 0.72, CI 0.094 to 1.815, P = 0.037) groups (Table 2). Table 2 shows the incidence of atelectasis after intubation (T1), after surgery (T2), and after 1h post-extubation (T3). There was no significant difference on the incidence of atelectasis at T1 between control groups (68%) and EIT groups (76%, OR 0.60, CI 0.192 to 1.816, P = 0.75). The difference was shown at T3 between these two groups. Atelectasis occurred in 23 in control group (80%) and 16 in EIT group (48%, OR 0.59, CI 0.149 to 2.215). In terms of lung ultrasound score, both lung consolidations and B-lines score were higher in control group than in EIT group at T2 (consolidations: 9 vs. 7, p = 0.027; B-lines: 11 vs. 8, P = 0.002) and T3 (consolidations: 7 vs. 4, P = 0.018; B-lines: 7 vs. 5, P = 0.037). Besides this, lung consolidations and B-lines score were lower at T3 (consolidations: 4; B-lines: 5) than at T1 (consolidations: 5; B-lines: 6) in the EIT group (Fig. 3).

A better lung compliance in the context of individualized PEEP was shown in the EIT group (20.0±3.3ml/cm H_2_O) compared with the control group using PEEP 5 (17.4±2.3 ml/cm H_2_O, P = 0.007) during anaesthesia (Table 3). Desaturation (pulse oximeter value below 95%) after extubation was observed in 7 in the control group and 1 in the EIT group (P = 0.048), one of which required treatment in control group. PEEP titration was well tolerated hemodynamically, there was no hemodynamic instability event in both groups.

## Discussion

In our study, we evaluated the effect of optimal PEEP titrated by EIT against fixed PEEP on perioperative atelectasis in children undergoing laparoscopic surgery. We found that an individualized PEEP could alleviate the degree of atelectasis and decrease the incidence of atelectasis.

Lung collapse is a frequent occurrence in anaesthetised children.^1^,^5^,^6^,^20^,^21^ The severity of atelectasis is directly proportional to its likelihood of impairing blood oxygenation, reducing lung compliance, inducing pneumonia, and delaying patient discharge.^22^,^23^ In recent years, great progress has been made to prevent perioperative atelectasis in differently validated ways. Whether using recruitment maneuvers, changing inspired fraction of oxygen, setting peep or methods aforementioned combined with each other are contribute to alleviate the degree of perioperative atelectasis.^5^,^9^ When it comes to PEEP, advantages such as maintenance of alveoli open, improvement of gas exchange, promotion of more homogenous ventilation and prevention of atelectasis could be understood.^7^ However, given individual differences (e.g., in children) and specific surgical factors (e.g., laparoscopic surgeries, operating position), a fixed PEEP may not always be optimal. In most intraoperative ventilator settings, an initial PEEP setting of 5 cm H_2_O is generally recommended.^24^ However, this may be insufficient for patients with pneumoperitoneum, potentially exacerbating atelectasis due to lung aeration impairment.^2^,^3^ A recent study showed that optimal PEEP varies between 5 to 12 cm H_2_O in children undergoing non-laparoscopic surgery with healthy lungs.^25^ When patients are exposed to excessive PEEP, hemodynamic impairments such as arterial hypotension or bradycardia may occur. Based on these factors, we chose a titration range of 3 to 15 cm H_2_O and found that a relatively higher individualized PEEP was used in children with pneumoperitoneum (Fig. 1).

EIT is a promising non-invasive bedside tool for optimal PEEP titration. It precisely provides the maximal lung overdistension and minimal atelectasis which means the optimal PEEP represents the best compromise between lung collapse and overdistension.^18^ Besides, EIT offers real-time visualization of regional lung volume changes. Our dynamic EIT images (Fig. 1) revealed a gradual decrease in the estimates of hyperdistension with the reduction of PEEP titration and an increase in the atelectasis area, predominantly in the dorsal region, which was in agreement with the findings of other authors who showed that individualized PEEP could improve the condition of lung aeration.^26^-^28^

Previous studies documented that setting individual PEEP applying EIT in adult patients under general anesthesia could improve oxygenation, reduce perioperative atelectasis.^29^,^30^ Notably, individualized PEEP appears more advantageous for patients undergoing laparoscopic as opposed to open surgery.^29^ In this study, we focused on children undergoing laparoscopic surgery and applied individualized PEEP exclusively during pneumoperitoneum in the EIT group. Our primary aim was to investigate the effect of optimal PEEP on pneumoperitoneum-induced atelectasis. We found a lower incidence of atelectasis in the EIT group at both T2 (64%, P = 0.037) and T3 (48%, P = 0.038) compared to the control group. Additionally, both the consolidation and B-lines scores were lower in the EIT group at T2 (consolidations: 9 vs. 7, P = 0.027; B-lines: 11 vs. 8, P = 0.002) and T3 (consolidations: 7 vs. 4, p = 0.018; B-lines: 7 vs. 5, P = 0.037), indicating the significant role of EIT in guiding PEEP titration in children and the efficacy of individualized PEEP in preventing pneumoperitoneum-induced atelectasis. Besides, optimal PEEP also improved the compliance in EIT group in our study.

Our study also had some limitations. First of all, we didn't analyze arterial blood gas because the operation time was short. Many studies have documented that individualized PEEP could improve oxygenation.^30^,^31^ Second, we only recorded the lung ultrasound score one hour after surgery to minimise trauma and hospital stay in laparoscopic hernia surgery in children. Consequently, we could not ascertain how long this kind of ventilation strategy for perioperative atelectasis prevention could persist. According to a previous study, whether aeration could fully restored 3h after extubation in children was irrelevant of the intra-operative ventilation strategy.^9^ Yet, in obese patients, ventilation distribution achieved through a RM and individual PEEP dissipated 2-6 hours post-extubation,^26^ implying a potential recurrence of atelectasis post-surgery. Nevertheless, both strategies demonstrated a positive impact on lung-protective ventilation. Future investigations should explore the role of individual PEEP throughout the perioperative period.

In conclusion, individualized PEEP titrated by EIT can mitigate lung collapse caused by capnoperitoneum, demonstrating good hemodynamic tolerance in children with normal pulmonary and cardiac function.

## Figures and Tables

**Table 1:** Characteristics of children

Variables	Control group (n=25)	EIT group (n=25)	P
Age (years)	4±1	4±1	0.420
Sex (male/female)	18/7	19/6	1.0
Height (cm)	108.9±12.0	107.2±11.4	0.606
Weight (kg)	19.8±4.5	19.3±3.5	0.697
Anaesthesia time (min)	57±14	55±13	0.665
Operation time (min)	29±8	29±3	0.859
Pneumoperitoneum time (min)	21±7	21±3	0.705

**Table 2:** Incidence of atelectasis in T1, T2 and T3

Characteristic	Control group (n=25)	EIT group (n=25)	P
T1 n (%)	17 (68)	19 (76)	0.754
T2 n (%)	23 (92)	16 (64)	0.037
T3 n (%)	20 (80)	12 (48)	0.038

T1: After intubation; T2: After sugery; T3: 1 hour in PACU (Postanesthesia care unit)

**Table 3:** Ventilatory and haemodynamic variables between groups.

Characteristic	Control group (n=25)	EIT group (n=25)	P
MAP (mmHg)	72.0±2.0	74.2±2.5	0.446
HR (beats/min)	107±4	104±2	0.603
Hemodynamic instability (n)	0	0	
Lung compliance (ml/cm H2O)	17.4±3.0	20.0±3.3	0.007
SpO2<95% in PACU (n)	7	1	0.048

Data are presented as mean±(SD) or absolute number of patients. HR, heart rate in beats per minute; MAP, mean arterial pressure; PACU, Postanesthesia care unit
